# Novel Insights into the Molecular Mechanisms Governing Embryonic Epicardium Formation

**DOI:** 10.3390/jcdd10110440

**Published:** 2023-10-24

**Authors:** Rita Carmona, Carmen López-Sánchez, Virginio Garcia-Martinez, Virginio Garcia-López, Ramón Muñoz-Chápuli, Estefanía Lozano-Velasco, Diego Franco

**Affiliations:** 1 Department of Human Anatomy, Legal Medicine and History of Science, Faculty of Medicine, University of Málaga, 29071 Málaga, Spain; rita@uma.es; 2 Department of Human Anatomy and Embryology, Faculty of Medicine and Health Sciences, Institute of Molecular Pathology Biomarkers, University of Extremadura, 06006 Badajoz, Spain; clopez@unex.es (C.L.-S.); virginio@unex.es (V.G.-M.); 3 Department of Medical and Surgical Therapeutics, Pharmacology Area, Faculty of Medicine and Health Sciences, University of Extremadura, 06006 Badajoz, Spain; garcialopez@unex.es; 4 Department of Animal Biology, Faculty of Science, University of Málaga, 29071 Málaga, Spain; chapuli@uma.es; 5 Cardiovascular Research Group, Department of Experimental Biology, University of Jaén, 23071 Jaén, Spain; evelasco@ujaen.es

**Keywords:** epicardium, cardiac development, transcriptional regulation, post-transcriptional regulation

## Abstract

The embryonic epicardium originates from the proepicardium, an extracardiac primordium constituted by a cluster of mesothelial cells. In early embryos, the embryonic epicardium is characterized by a squamous cell epithelium resting on the myocardium surface. Subsequently, it invades the subepicardial space and thereafter the embryonic myocardium by means of an epithelial–mesenchymal transition. Within the myocardium, epicardial-derived cells present multilineage potential, later differentiating into smooth muscle cells and contributing both to coronary vasculature and cardiac fibroblasts in the mature heart. Over the last decades, we have progressively increased our understanding of those cellular and molecular mechanisms driving proepicardial/embryonic epicardium formation. This study provides a state-of-the-art review of the transcriptional and emerging post-transcriptional mechanisms involved in the formation and differentiation of the embryonic epicardium.

## 1. Origin of the Embryonic Epicardium

During cardiac development, the epicardium originates from an extracardiac primordium, the proepicardium (PE), which is constituted by a cluster of mesothelial cells located on the cephalic and ventral surfaces of the liver-*sinus venosus* limit in avian embryos [1,2,3,4,5,6,7,8] and the pericardial side of the *septum transversum* in mammalian embryos [9]. In early embryos, the epicardium acquires the form of a squamous cell epithelium that either rests directly on the surface of the myocardium or covers a subepicardial space that appears to be densely populated by mesenchymal cells [10]. It is also assumed that the epicardium is not a simple mesothelium but that it is made up of discrete clusters of heterogeneous cell types which include a hematopoietic contribution from distinct origins, encased by an extracellular matrix (ECM) that anatomically resembles a stem or progenitor cell ‘niche’ [11].

It was reported that PE originates in the periphery of the heart forming fields in the lateral plate mesoderm (LPM) as part of an early cardiac progenitor lineage [12]. A single PE bud is formed during zebrafish cardiogenesis [13] while in other fish, such as the sturgeons, bilateral primordia are formed which subsequently converge into a single PE structure in the embryonic midline [14]. Noticeably, in mice, bilateral PE anlage is also established and further develops similarly to sturgeons while in chick embryos only the right-side anlage develops [15]. Interestingly, chicken PE arises both from the splanchnic layer of the LPM and the somatic mesoderm, which also contributes to the mesothelial portion of the PE that forms the typical villous protrusions [16,17,18]. The above observations suggest that the embryonic left–right signal might play a significant role during PE development. Furthermore, while all proepicardial cells in a given species (e.g., in mice, zebrafish, and chicks) are morphologically similar, they exhibit a distinct differentiation potential due to various marker expressions [19,20,21]. Therefore, the detailed composition of the embryonic epicardium is not well known.

**Figure 1:**
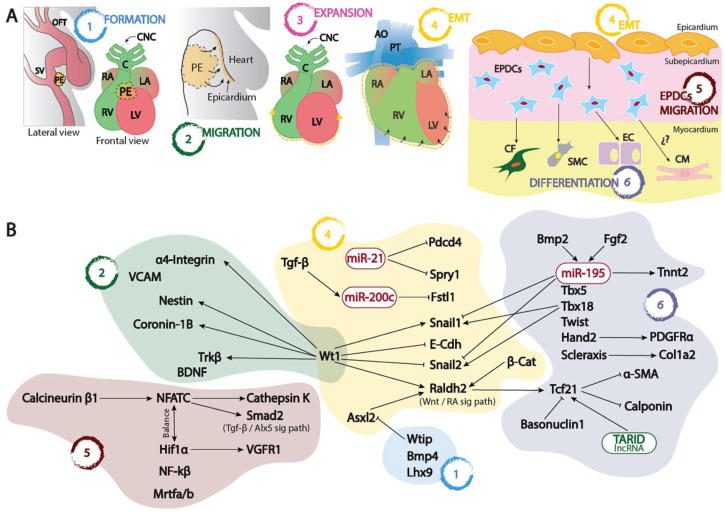
(**A**) Schematic representation of the proepicardium (PE) and embryonic epicardium formation since its origin at the *sinus venosus*–*septum transversum* (1), its migration (2) and expansion (3) into the naked myocardium, its epithelial to mesenchymal transition leading to the formation of the EPCDs (4), and finally the migration and invasion (5) of the embryonic myocardium differentiating into distinct cell types (6). (**B**) Schematic representation of the distinct transcriptional and post-transcriptional regulatory mechanisms involved in each of the distinct processes depicted in (**A**). OFT, outflow tract; SV, sinus venosus; PE, proepicardium; RA, right atrium; LA, left atrium; RV, right ventricle; LV, left ventricle; CNC, cardiac neural crest, C, conus; AO, aorta; PT, pulmonary trunk; EPDCs, epicardial derived cells; CF, cardiac fibroblasts; SMC, smooth muscle cells; EC, endothelial cells; CM, cardiomyocytes; EMT, epithelial to mesenchymal transition.

After PE formation, cells translocate to the myocardial surface of the looping heart, where they adhere, migrate, and proliferate to form a squamous epithelial layer: the embryonic epicardium [22]. It has been described that PE translocation to the myocardium takes place through distinct mechanisms among species, including direct contact and/or the release of free-floating cell clusters (or cysts) into the pericardial cavity or PE cell migration from the *sinus venosus* towards the heart along the surface of the inflow tract [23,24,25,26]. After attachment to the myocardial surface, the cells start to migrate laterally until the complete heart is enveloped by the epicardium [5]. It has been described that the epicardium surrounding the arterial pole does not originate from the PE but from the coelomic/pericardial mesothelium at the area where the aortic sac leaves the pericardial cavity [27,28]. This cell population will contribute to the outer mesenchymal layer of the arterial pole within the pericardial cavity, contributing to the arterial epicardium formation, whereas the PE-derived epicardium will cover the myocardial outflow tract (Figure 1A).

## 2. Derivates of the Embryonic Epicardium

Once the epicardium is established, epicardial cells will be directly involved in the formation of the myocardium. A group of epithelial cells will undergo an epithelial–mesenchymal transition (EMT), giving rise to epicardium-derived cells (EPDCs), and then migrate into the matrix in the subepicardial layer to form the subepicardium [22]. The subepicardium thickness will eventually vary according to the underlying heart structure to be covered and may vary among species. In particular, in chick embryos, the subepicardium is relatively thin in the atrial and ventricular myocardium. However, in the atrioventricular sulcus, the subepicardium is thicker in order to provide those EPDCs needed for coronary formation [29,30].

From the subepicardium, mesenchymal EPDCs will form migratory processes and invade the myocardium in a spatio-temporarily regulated fashion where several factors expressed in the underlying myocardium will define the permissiveness for EPDCs. These migrate into the underlying myocardium in a tangential pattern and most of them are retained directly underneath the local area of the subepicardium [5,30,31].

Within the myocardium, EPDCs present multi-lineage potential, differentiating into smooth muscle cells (SMCs) and contributing to the coronary vasculature and cardiac fibroblasts (CF) of the mature heart [22,26] in line with reports linking EMT and the acquisition of stem cell properties [32]. At present, it is still controversial as to whether these distinct cell lineages are already established in the proepicardium or if EPDCs acquire pluripotentiality during or after EMT [33].

Most EPDCs reach their final positions (i) around the coronary arteries as smooth muscle cells (SMCs) and adventitial fibroblasts [27,29,34,35,36]; (ii) in the atrioventricular cushions [27,31,35]; (iii) in the subendocardium of the ventricular trabeculae and atria [27,30,31]; and (iv) in the ventricular myocardium as interstitial fibroblasts [31]. Other contributions of EPDCs to cardiac endothelial cells (ECs) [37,38,39,40] and cardiomyocytes (CMs) [41,42] have also been described although this issue needs further research [43,44]. Therefore, both the *sinus venosus* and ventricular endocardium are considered major contributors to ECs [45] while EPDCs have a low contribution, if any [46]. With respect to epicardial-derived cardiomyocytes, lineage tracing studies, by using Scleraxis, WT1, and TBX18, have indicated possible epicardial-derived cardiomyocyte labeling, although their contribution is still controversial [41,43,44].

Additionally, after the embryonic epicardium has covered the developing heart, the epicardial cells and the EPDCs will produce cytokines and growth factors in order to induce the myocardial development [25]. These factors and the regulation of their expression are still rather unknown but a number of Fgfs, particularly Fgf-9 and Igf-2, have been proposed to be mitogenic factors for the developing cardiomyocytes [47,48,49]. In this sense, impaired embryonic epicardium development and/or cytokines and growth factor delivery results in deficient ventricular chamber maturation [47,50,51,52,53,54].

In contrast to the embryonic epicardium, the postnatal mammalian epicardium seems to be a dormant single-cell layer since most genes involved in epicardial activation, such as WT1, Tbx18, and Raldh2 are rapidly downregulated postnatally, being scarcely detectable only during the first three months in mice [55]. This is an interesting issue since regeneration of the injured myocardium in non-mammalian species is dependent on epicardial activation and re-expression of genes characteristic of the embryonic epicardium [56]. Even the murine heart, which has a very limited regenerative potential, shows a re-expression of the embryonic epicardial gene program and generation of Wt1-lineage positive subepicardial mesenchyme after myocardial infarction [57].

## 3. Transcriptional Regulation of the Embryonic Epicardium

The development of the embryonic epicardium and its cellular derivatives is a highly complex and regulated process. In the former section, the crucial importance of the epicardial derivatives for both the constitution of the fibrous skeleton of the heart and its vascularization was shown. Furthermore, the epicardium and EPDCs are the origin of molecular signals towards the developing myocardium. The correct growth and compaction of the cardiac wall depend on these pathways. Thus, the precise regulation of these cellular and molecular mechanisms requires a precise orchestration of a set of transcription factors in order to activate or inhibit the genes involved in all these processes.

Transcription factors act at different levels during the development of the epicardium and its derivatives [58]. We can distinguish the development of the proepicardium and the migration of epicardial cells over the myocardium; the EMT giving rise to EPDCs, these EPDCs differentiate mainly into fibroblasts and vascular SMCs, and the epicardial derivatives invade into the myocardial wall. We will describe the main transcription factors involved in the control of all these processes below.

### 3.1. Proepicardial Development

As we have described above, the proepicardium develops in the ventral side of the venous pole of the heart. Little is known about the molecular mechanisms governing the location and development of this cluster of cells that expresses most of the characteristic epicardial genes. Liver-derived signals induce ectopic expression of proepicardial markers in chick embryos [59] and this can explain the proximity of the proepicardial bud to the developing liver. BMP4 signaling has also been proposed as a proepicardial inductor [15,60]. In *Xenopus*, the LIM homeodomain protein Lhx9 is essential for the correct position of the proepicardial organ on the *septum transversum* [61]. Its deficiency leads to proepicardial malposition and loss of attachment to the heart surface.

The hypothesis of the evolutionary origin of the proepicardium from an ancestral external glomerulus should also be considered in order to explain the mechanisms of its development [62,63]. For example, Wilms’ tumor 1 interacting protein (WTIP), an interacting partner of Wilms’ tumor protein (WT1) essential for the development of podocytes, is also necessary for PE specification in the zebrafish heart. Overexpression of WTIP mRNA induces ectopic expression of PE markers in the cardiac and pharyngeal arch regions [64].

### 3.2. Epicardial Migration and Epicardial EMT

The function of the Wilms tumor suppressor gene *Wt1* was originally associated with the development of the kidneys and gonads [65]. However, its importance for epicardial development was soon evidenced. WT1, acting as a transcriptional activator or repressor, is involved in a number of genetic mechanisms leading to the transformation of the epicardium into EPDCs (reviewed in [66]). WT1 promotes the expression of integrin α4, a receptor for myocardial vascular cell adhesion molecule (VCAM). This regulates adhesion of the epicardial cells to the myocardial wall and their migration. Other targets of WT1 are nestin (an intermediate filament) and coronin-1B (an actin-binding protein), related to cell motility and migration [67,68]. WT1 activates expression of TrkB, the receptor of BDNF, involved in coronary vascularization [69]. But the main processes activated by WT1 in the epicardium are directly related to the core mechanisms of EMT. These are the Snail/E-cadherin and the Wnt/retinoic acid pathways.

WT1 is a direct transcriptional activator of Snail1 and a repressor of E-cadherin in mice [70]. Snail1 plays a pivotal role in the regulation of EMT in mammals through repression of epithelial genes and activation of genes related with mesenchymal phenotype and cell motility. Repression of E-cadherin also leads to loss of the epithelial phenotype. However, it has been shown that Snail1 silencing in the epicardium does not impair epicardial EMT [71], suggesting that this process can be activated by different means. In fact, Snail2 has also been suggested to be involved in the EMT of the murine epicardial cells, although in the mouse this transcription factor is activated by Tbx18 and inhibited by WT1 [72]. On the other hand, there is evidence of the importance of Snail1 in avian epicardial EMT [73]. Thus, the roles of Snail1 and Snail2, as direct executors of the epicardial EMT, in repressing the epithelial phenotype and promoting a mesenchymal phenotype need further investigation.

Retinoic acid and the canonical Wnt pathway have also been shown to be involved in the epicardial EMT. WT1 has a significant role in the retinoic acid signaling pathway since it promotes the epicardial expression of RALDH2, a key retinoic acid-synthesizing enzyme highly expressed in the epicardium [74]. Retinoic acid promotes a cytoskeletal rearrangement in epicardial cells necessary for EMT in a RhoA-dependent fashion [75]. Systemic loss of the nuclear receptor αRXR leads to malformation of the epicardium and deficient EMT [76]. Conditional deletion of this receptor in the epicardium causes a similar phenotype [52]. These authors identified a retinoid-dependent *Wnt* signaling pathway cooperating in the epicardial EMT. In fact, β-catenin is essential for this process [77]. Thus, in parallel with the role played by WT1 in the control of Snail1 and E-cadherin expression, WT1 regulates epicardial EMT through canonical Wnt, non-canonical Wnt, and retinoic acid signaling pathways [78].

The retinoic acid signaling pathway must be carefully regulated both positively and negatively for correct epicardial and coronary vessel development [75]. The above-mentioned factor WTIP, a WT1 partner expressed in the proepicardium and epicardium, may be relevant in the inhibition of this pathway. WTIP blocks ASXL2, a chromatin factor highly expressed in the heart that promotes retinoic acid signaling [79].

### 3.3. Invading the Myocardium

A key event in the epicardial contribution to heart development is the invasion of the myocardial wall by the EPDCs. This is necessary in order to establish the fibrous skeleton of the heart and to organize the complex vascularization of the myocardium. The transcription factor NFATC1 becomes activated and moves to the nucleus when it is dephosphorylated by calcineurin. NFATC1 is required for myocardial invasion of EPDCs by induction of cathepsin K expression, an enzyme that degrades extracellular matrix. Loss of NFATc in murine EPDCs causes loss of cathepsin K expression in the myocardial interstitium and embryonic death. The mutant embryos show reduced coronary vessel and fibrous matrix penetration into myocardium [80]. Conditional depletion of calcineurin b1 (a NFAT activator) in the epicardium also induces defects in the coronary smooth muscle [81]. This study also showed a direct role for NFATc in the transcription of Smad2, another transcription factor necessary for transduction of TGFβ-Alk5 signaling.

The hypoxia inducible transcription factor-1α (HIF-1α) seems to be a negative regulator of the myocardial invasion by EPDCs. The expression of constitutively active HIF-1α in the embryonic avian epicardium reduced EPDC migration into the myocardium, probably through upregulation of the VEGFR1, a decoy receptor that sequesters VEGFR [82]. A balance between signals promoting and inhibiting myocardial invasion by EPDCs may be necessary for correct patterning of the coronary vasculature.

The protein NFκB plays also a role in the acquisition of the invasive ability of epicardial cells. Epicardial cells incubated with an inhibitor of NF-kB signaling cannot invade a collagen gel in response to TGFβ2 or BMP2. TGFβR3-null mice fail to activate the NFκB pathway in the epicardium and show reduction in EPDCs and coronary vascularization [83].

Finally, the myocardin-related transcription factors MRTF-A and B have been associated with the control of EPDC motility and the activation of the migratory and invasive program. Conditional ablation of MRTF in the epicardium leads to decreased migration of EPDCs and subepicardial hemorrhage due to the depletion of pericytes, a cell type derived from the EPDCs in an MRTF-dependent process [84].

### 3.4. Differentiation of EPDCs

The EPDCs mainly contribute to fibroblasts and coronary smooth muscle. As we stated above, it is uncertain as to whether different lineages of EPDCs are already established in the proepicardium or if the EPDCs are multipotent and differentiate in response to local cues [21,85]. A number of transcription factors, mainly belonging to the bHLH family, are critically involved in the control of their differentiation.

The bHLH protein TCF21 (aka epicardin, POD1, or capsulin) is required for normal epicardial development and it regulates EPDC differentiation (reviewed in [86]). TCF21 loss of function leads to the premature differentiation of EPDCs. TCF21 heterodimerizes with E12, another bHLH factor, for repression of transcription. In the cardiac interstitium, downregulation of TCF21 leads to the differentiation of EPDCs into smooth muscle while persistence of its expression promotes fibroblast identity. Although targets of TCF21 have not been identified, in mesenchymal cells the smooth muscle markers SM22a, calponin, and αSMA are targets repressed by TCF21, suggesting a similar role in EPDCs. Interestingly, retinoic acid activates the expression of TCF21, thus keeping the EPDCs in an undifferentiated state [87]. This repressor role of TCF21 on the EPDCs has also been demonstrated in *Xenopus* [88]. Loss of TCF21 function in zebrafish also reduces FGF and VEGF signaling in the heart, reducing myocardial growth [89].

The epicardial expression of TCF21 is negatively regulated by basonuclin-1, a zinc-finger transcription factor, and this can be related to the relative proportion of differentiated fibroblasts and smooth muscle cells. Loss of basonuclin-1 in epicardium derived from human pluripotent stem cells leads to a predominance of TCF21+ cells and a reduction in smooth muscle progenitors [90].

The T-Box transcription factor Tbx18 also has a role in maintaining the progenitor status of EPDCs, similar to that described above for TCF21. The role of Tbx18 in murine epicardial EMT is controversial; it may induce the process by activation of Snail2 [72] but other studies consider Tbx18 dispensable for epicardial EMT [91,92]. However, Tbx18 has a critical role in preventing the premature differentiation of smooth muscle cells. Tbx18-null mice show defects in the remodeling of the coronary vascular plexus and alteration in the expression of genes related to vascularization [91,92]. Hypoxia causes upregulation of Tbx18 in the epicardium, inducing the expression of Snail1 and enhancing EMT and motility [93]. Another T-Box factor, Tbx5, probably also plays a role in epicardial development since its deletion in the proepicardium leads to decreased production of EPDCs, reduced migration into the myocardial wall, and low density of the coronary vessels [94].

Other bHLH transcription factors are involved in the origin and differentiation of EPDCs. For example, Twist1 is expressed in EPDCs of avian embryos and it promotes mesenchymal cell proliferation and migration [95]. Scleraxis is expressed in a proepicardial subdomain and during the early stages of the epicardium development. The loss of function in mice leads to persistent expression of EMT markers, suggesting a role in the differentiation of EPDCs, mainly towards fibroblasts [96]. In fact, Scleraxis induces the expression of Col1a2 in adult cardiac fibroblasts [97]. Finally, Hand2 is necessary for normal development of the epicardium where it activates PDGFRα [98]. This receptor of PDGF is required for epicardial EMT and the differentiation of EPDCs [99].

### 3.5. Other Transcription Factors Involved in Epicardial Development

A number of transcription factors were recently identified as participating in different phases of epicardial development. For example, the SRY-box protein Sox9 is expressed in EPDCs and must play some role in epicardial EMT since its overexpression rescues the defective EMT caused by PDGF receptor ablation in the epicardium [99]. This role appears to be dispensable since Sox9 loss of function has no effect on this process.

The epicardial deletion of GATA4 and GATA6 leads to a drastic reduction in the number of coronary endothelial cells [100]. The replacement of GATA4 by GATA6 in the systemic GATA4-null mice does not rescue the epicardial phenotype suggesting that they do not play redundant roles in this tissue [101].

CDX1, a caudal-related family member, has recently been shown to be involved in epicardial EMT. Its expression promotes EMT but a low dose of CDX1 is required for enhanced migration and differentiation of EPDCs into vascular smooth muscle. Both continued high-level expression of CDX1 and CDX1 deficiency reduce the ability of EPDCs to migrate and differentiate [102].

The kinases LATS1 and LATS2 are important regulators of cell fate. Epicardial deletion of LATS1/2 in mice embryos is lethal due to defective coronary artery remodeling. These kinases inhibit the transcriptional function of the factor YAP, a Hippo pathway effector that prevents EPDCs differentiation into fibroblasts [103]. In fact, YAP inhibition reduces proliferation in EPDCs [104]. These studies reveal the involvement of the Hippo signaling pathway in the regulation of EPDC differentiation.

Finally, TFEB, a member of the microphthalmia-associated transcription factor family has been involved in the negative regulation of the epicardial EMT by activation of the TGIF1 promoter. TGIF1 (thymine–guanine-interacting factor 1) is a repressor of the TGFβ/SMAD signaling pathway [105]. Thus, epicardial overexpression of TFEB is lethal due to defective EMT.

### 3.6. Post-Transcriptional Control of Epicardial Development

Transcriptional regulation is the main molecular mechanism driving cell specification and determination during embryonic development. The identification of novel players, i.e., non-coding RNAs, that modulate post-transcriptional gene regulation has added an additional layer of complexity to the understanding of the molecular mechanisms driving these morphogenetic processes. Non-coding RNAs are currently classified according to their length into two subclasses: (a) small non-coding RNAs (<200 nt) including piwiRNAs, microRNAs, and snoRNAs, among others, and (b) long non-coding RNAs (>200 nt), including lncRNAs and circRNAs [106,107]. microRNAs represent the most abundant and well-studied class of small non-coding RNAs. microRNAs are nuclearly encoded and transcribed and then exported to the cytoplasm when maturation occurs. Mature microRNAs exert their function by base-pair complementarity with target transcripts leading to RNA instability and/or translation blockage [108]. On the other hand, lncRNAs are also nuclearly transcribed but they can exert their function both within the nucleus as well as in the cytoplasm [109,110]. Finally, circular RNAs (circRNAs) are normally generated by exon–exon back-splicing and they have been found in a wide range of eukaryotic species, exerting a variety of biological functions, upon which their function as microRNAs sponges is particularly relevant [111].

Multiple forms of evidence have demonstrated that non-coding RNAs, including both microRNAs and lncRNAs, display differential expression profiles in homeostasis and pathological conditions [112,113,114]. Within the cardiovascular field, multiple studies have demonstrated tissue-specific expression in both normal and pathological conditions [115,116,117] as well as during cardiac development and regeneration [118,119,120,121]. Similarly, ample evidence is available about the functional role of distinct microRNAs an lncRNAs during both cardiogenesis as well as in distinct pathophysiological conditions, such as cardiac structural and arrhythmogenic diseases [122,123,124,125,126]. However, in the context of PE and epicardial development, limited information is yet available.

The first evidence on the functional role of microRNAs was reported by the seminal work of Singh et al. [127], demonstrating that conditional deletion of *Dicer*, a key microRNA processing exonuclease in the developing epicardium, was essential for the correct development of the coronary vessels in mice. Subsequently, several studies have provided additional evidence on the role of microRNAs in key epicardial-derived biological processes such as the epithelial—mesenchymal transition (EMT) [128,129,130], cardiac tissue repair [131,132,133,134,135], and cardiomyocyte proliferation [134,135].

EMT is required for the colonization of the embryonic subepicardial space emanating from the nascent embryonic epicardial layer, leading to the formation of EPDCs. Bronnum et al. [136] identified miR-21 as a key microRNA regulating Pdcd4 and Spry1 and thus controlling fibrogenic EMT while, more recently, Pontemezzo et al. [137] reported that Tgf-β1 induced EMT resulted in miR-200c inhibition that, in turn, modulated *Fstl1* impacting on the mouse epicardial cell transition.

Epicardial deployment is essential for myocardial wall growth, as indicated in previous sections. The absence or impaired development of the epicardial layer results in a thin myocardium and reduced compact myocardium development [138]. Jang et al. [139] recently reported that HDAC3 regulation of miR-322 and miR-503 in the epicardial layer is essential for the modulation of two distinct growth factors, i.e., Igf2 and Fgf9, that, if impaired, restrict myocardial growth.

Activation of the epicardial layer is required for cardiac repair in different species. Particularly, an eventual recovery of the embryonic potential by the adult epicardium remains an interesting approach, as stated above. This recovery could allow the production of cells, cytokines, or growth factors competent for cardiac repair. In fact, proepicardial cells, if cultured in isolation, can spontaneously generate beating cardiomyocytes, a process that is promoted by Bmp and halted by Fgf signaling [140]. Furthermore, adult epicardial cells, if primed with thymosin β4, can differentiate into mature and fully functional integrated cardiomyocytes in adverse conditions, i.e., in myocardial infarction, yet in a very limited yield [55]. All these studies nonetheless support the notion of the key role of epicardial cells in cardiac regeneration. Dueñas et al. [141] demonstrated that Bmp and Fgf regulated miR-195 expression and furthermore, that miR-195 administration could promote PE to enhance cardiomyogenesis, a process modulated by Smurf1 and Smad3 in chicken explants. More recently, Garcia-Padilla et al. [142] reported that while Bmp and Fgf signaling could similarly modulate miR-195 expression in mouse PE explants, they failed to augment cardiomyogenesis, thus reporting species–specific differences in this microRNA-modulated pathway.

Cardiomyocyte proliferation represents a key biological cornerstone to healing the broken heart. Diverse experiments in different species have reported an essential role of the epicardium in promoting cardiomyocyte proliferation in several cardiac injury models [90,143,144,145]. Del Campo et al. [146] reported that epicardial-derived extracellular vesicles (EVs) loaded with cardiomyogenic reparative microRNAs, i.e., miR-30a, miR-30e, miR-27a, and miR-100, were capable of inducing cardiomyocyte cell cycle reentry in a mouse model of myocardial infarction. Similarly, and more recently, Zhu et al. [147] reported that lncRNA TARID could modulate Tcf21 transcription faction function in EPDCs leading to improved cardiac function in a mouse model of myocardial infarction.

In summary, these studies reported the emerging role of non-coding RNAs in modulating key biological processes orchestrated by or with the contribution of the epicardium. In coming years, we will witness increasing evidence of the functional role of these types as well as of other types of non-coding RNAs, therein including lncRNAs and circRNAs in epicardial development in homeostasis and disease.

## 4. Conclusions and Perspectives

For a long time, it was believed that the epicardium derived from the outer layer of the embryonic heart and so it was labeled as ‘epimyocardium’. However, more recent studies have revealed an extracardiac origin of the epicardium, derived from a proepicardium located at the caudal end of the cardiac tube, together with evidence of an epicardial EMT process, opening a new highly productive research field. We now understand that a major portion of cardiac cells, primarily coronary smooth muscle and fibroblasts, originate from the embryonic epicardium. There is also evidence suggesting the existence of a minor contribution of EPDCs to other cell types, such as coronary endothelium. In addition to this large-scale cellular contribution, the epicardium and its mesenchymal derivatives play a crucial signaling role in myocardial growth and maturation. Therefore, the role of the epicardium is fundamental for cardiac morphogenesis, implying complex molecular mechanisms of control and regulation. As described in this review, numerous signaling and regulatory pathways, both at transcriptional and post-transcriptional levels, were uncovered in the last three decades (Figure 1B).

Despite the present knowledge of the epicardial contribution to cardiac development, many aspects still remain unclear. In particular, it is not well known as to whether different derivatives from the embryonic epicardium belong to distinct cell lineages established in the proepicardium or that, alternatively, EPDCs are in fact pluripotential (see for a recent review [33]). The signaling mechanisms between the epicardium/EPDCs and the embryonic myocardium are not fully understood yet. Noticeably, the importance of small and long non-coding RNAs in epicardium and EPDC development is just beginning to be fruitful. Is the adult epicardium capable of recovering the potential to acquire embryonic features? What is more, would it be able to transdifferentiate or produce signals for cardiac repair?

These and other questions related to the epicardium will continue to drive research on the embryonic epicardium and its crucial roles in cardiac morphogenesis and regeneration.
